# Influence of Bilateral Subthalamic Nucleus Deep Brain Stimulation on the Lipid Profile in Patients With Parkinson's Disease

**DOI:** 10.3389/fneur.2020.563445

**Published:** 2020-10-09

**Authors:** Joanna Samborska-Ćwik, Stanisław Szlufik, Andrzej Friedman, Tomasz Mandat, Andrzej Przybyszewski, Dariusz Koziorowski

**Affiliations:** ^1^Department of Neurology, Faculty of Health Science, Medical University of Warsaw, Warsaw, Poland; ^2^Department of Neurosurgery, Maria Sklodowska-Curie National Research Institute of Oncology, Warsaw, Poland; ^3^Department of Informatics, Polish-Japanese Academy of Information Technology, Warsaw, Poland

**Keywords:** deep brain simulation, Parkinson's disease, lipids, cholesterol, triglycerides, body weight

## Abstract

**Background:** Subthalamic nucleus deep brain stimulation (STN-DBS) is a valuable alternative to pharmacotherapy alone in an advanced Parkinson's disease (PD). Given the growing number of patients with STN-DBS, its impact on the comorbidities should be considered.

**Aim:** The aim of this study was to evaluate the influence of bilateral STN-DBS on the lipid profile in patients with PD.

**Methods:** Three groups of parkinsonian patients were included: 20 treated pharmacologically–PHT group, 20 newly qualified for STN-DBS–DBS group, and 14 postoperative patients (median 30 months after surgery)–POP group. Plasma concentrations of the total cholesterol (TC), low-density lipoprotein cholesterol (LDL-C), high-density lipoprotein cholesterol (HDL-C), triglycerides (TG), and body weight were measured thrice in 9 ± 2 month intervals.

**Results:** A significant increase in the LDL-C concentration is observed early after surgery in the DBS group (11.4 mg/dl, *P* < 0.01) followed by adverse changes in the HDL-C (−7.7 mg/dl, *P* = 0.01) and TG (14.1 mg/dl, *P* = 0.05) plasma levels. In the POP group, the average level of TC at the first visit was significantly higher (*P* < 0.01) than in the other groups and the TG level was higher than in the PHT group during the follow-up (*P* < 0.01). A strong positive correlation with body weight alteration after surgery was observed only for long-term changes in the TG levels.

**Conclusions:** Our data indicate that STN-DBS may negatively affect the cardiometabolic profile of patients. Similarly to body weight gain, an increase in the LDL-C concentration occurred early after surgery while adverse changes in the HDL-C and TG plasma levels were more gradual.

## Introduction

Deep brain stimulation (DBS) represents a well-known treatment of Parkinson's disease, being a favorable option for patients whose symptoms cannot be adequately controlled with medications (1). Although the positive impact of bilateral subthalamic nucleus deep brain stimulation (STN-DBS) on motor symptoms and reduction of the dose-related side effects of levodopa is unquestionable (2, 3), its influence on non-motor symptoms and comorbidity requires further investigation.

A frequently observed side effect of DBS is weight gain after the implantation of electrodes (4, 5). It is a reversal of the tendency observed among patients not treated surgically as numerous studies reported the unintentional continuous weight loss in the natural course of the disease, as summarized by van der Marck et al. (6). According to inter alia Adams et al. (7), patients lose weight regardless of the antiparkinsonian drugs intake. Others, like Pålhagen (8), suggests that levodopa *per se* contributes to weight loss. Various reports indicate that weight gain after DBS is more than a homeostatic reaction compensating the previous weight loss and may result in overweight or obesity (4, 9).

The influence of DBS on other cardiometabolic risk factors has been far less explored.

Many clinical observations imply a favorable cardiometabolic profile in patients with Parkinson's disease (especially levodopa-treated) compared to the general population (10–12). Various authors reported lower levels of low-density lipoprotein cholesterol (LDL-C), triglycerides (TG), total cholesterol (TC), very low density lipoprotein cholesterol, and apolipoprotein B in PD patients than in healthy individuals (12–14). Cassani et al. (10) observed a positive correlation between the high-density lipoprotein cholesterol (HDL-C) level and disease duration. Moreover, Scigliano et al. (11) imply that levodopa-treated patients have a significantly lower plasma levels of TG and TC in comparison to untreated patients, which might be attributed to the inhibitory effect of levodopa-derived dopamine on the sympathetic nervous system. However, very few studies refer to the lipid profile changes after DBS (15). It may be supposed that the weight gain after surgery should lead to negative changes in other cardiometabolic parameters. On the other hand, changes in the lipid profile in PD patients seem to be independent of the body composition or nutritional state (10, 12), which additionally substantiate exploration of other factors (such as treatment of Parkinson's disease *per se*) affecting the biochemical markers in this population of patients.

Therefore, we decided to conduct a study evaluating the possible impact of STN-DBS on the serum lipids in patients with PD.

## Materials and Methods

### Participants and Methods

Data was collected from 54 patients (29 males, 25 females) aged from 31 to 79 years old with a clinical diagnosis of idiopathic Parkinson's disease. All patients met the UK Parkinson's Disease Society Brain Bank criteria (16) and CAPSIT-PD criteria (17) to fulfill the qualification criteria for a bilateral STN-DBS implantation. No individuals suffered from additional neurological conditions.

The study population was composed of three groups (marked, respectively, as PHT, POP, and DBS) according to the method of treatment. The PHT (pharmacotherapy) group consisted of 20 patients (9 males, 11 females) treated with the medication only. The DBS group included 20 patients newly qualified for STN-DBS (12 males, 8 females). The POP (postoperative) group recruited from patients with a medical history of such surgery–median 30 months prior to inclusion in the study (*N* = 14, 8 males, 6 females).

PHT patients received the optimal pharmacotherapy alone (levodopa/dopamine agonist in monotherapy or coadministered, or/and combined with selegiline or amantadine) for the whole study duration.

Patients from the other groups underwent the surgical procedure based on the MRI, stereotactic contrast-CT, and electrophysiological mapping to precisely locate the electrodes (3389-28, Medtronic, Minneapolis, MN) in the area of STN in both hemispheres. Later, the electrodes were connected to the internal pulse generators (Activa SC, Medtronic, Minneapolis, MN) which were placed in the subcutaneous tissue in the subclavian region. Stimulation settings and pharmacotherapy were adjusted postoperatively to obtain the best motor efficacy of chronic stimulation and reduce levodopa-related dyskinesia and fluctuations. Patients from the DBS and POP groups received also similar medications (in various combinations) as the PHT patients.

Each patient was examined thrice—at the time of inclusion (V1) and in 9 ± 2-month intervals (V2, V3). In the case of the DBS group, the first visit was prior to surgery and the following two after the implantation of electrodes. For the POP group, all three visits took place after the surgery.

Each time, a thorough neurological examination and assessment of the severity of symptoms against the UPDRS (Unified Parkinson's Disease Rating Scale) and Hoehn-Yahr was performed by the same neurologist experienced in motor disorders. The efficacy of the treatment was evaluated inter alia by a comparison of the UPDRS scores during “on” and “off” phases, where “on” means the normal antiparkinsonian medication intake and, in the case of the surgically treated patients, with the stimulator switched on, and “off” means at least 12 h withdrawal of levodopa and 24-h without other antiparkinsonian drugs and, for the postoperative assessment, both stimulators also switched off for at least half an hour.

Fasting plasma concentrations of the TC, LDL-C, HDL-C, and TG were quantified at the Clinical Laboratory of Masovian Brodnowski Hospital (Alinity CI system, Abbott) during the subsequent visits. Current body weight was measured to the nearest 1 kg and its proportionate inter-visit gain was calculated.

Except for several patients in the POP group receiving statins after the first assessment (as explained in the Results section), no other medications that are known to affect the lipid profile were taken by our patients. Patients reported no radical voluntary changes in their diet or lifestyle during the study.

The main analysis concerned the DBS group, in which the pre-surgery data was also collected, with the PHT patients as the control. Inclusion of the additional group (POP) enabled a preliminary assessment of the longer term effects of STN-DBS; however no pre-surgery data was available in those patients. Groups were similarly distributed in terms of sex, age, and disease duration. The general characteristics of the study population are presented in Table 1.

**Table 1:** Baseline characteristics of the study population at the time of inclusion.

	**PHT group**	**DBS group**	**POP group**
Number of patients (male/female)	20 (9/11)	20 (12/8)	14 (8/6)
Age	59.1 ± 11.7	55.2 ± 8.6	51.4 ± 8.7
Duration of the symptoms [years]	10.4 ± 4.9	11.3 ± 3.9	13.2 ± 3.5
UPDRS “on” / “off”	25.4 ± 9.7/50.9 ± 16.1	28.3 ± 11.6/59.7 ± 14.7	20.4 ± 11.6/61.2 ± 17.2^*^
UPDRS part III “on” / “off”	12.8 ± 4.8/32.3 ± 10.5	11.5 ± 5.5/34.1 ± 7.9	10.2 ± 6.5/39.5 ± 12.4^*^

*The groups do not vary significantly (P > 0.05)*.

* *In the POP group “on” – on medication + on stimulation and “off” – off medication + off stimulation*.

The study was approved by the Ethics Committee of Medical University of Warsaw. The experiments were conducted in accordance with the ethical standards of the Declaration of Helsinki. All participants gave informed consent prior to their inclusion.

### Statistical Analysis

In order to establish the inter-group effect on the results obtained by the patients, a linear mixed model analysis was implemented through the use of the LME4 (version 1.1) with intercepts for subjects included as random effects. Pairwise interactions between each fixed factor were included in the model. Tukey contrasts (from lsmeans package, version 2.25) were used to compare the results between time points and treatments. Non-parametric tests including the Mann–Whitney *U*-test and Wilcoxon signed-ranked test were carried out to estimate the inter-group and inter-visit variation, respectively, for the non-normally distributed data. The Fisher exact test was applied to compare the categorical variables. Correlations between the biochemical parameters, body weight, and symptoms severity were estimated using the Pearson or Spearman correlation tests when appropriate. All calculations were performed using the R statistical computing software (version 3.3) and STATISTICA (version 13.1). Continuous variables are reported as means ± SD. *P* < 0.05 were considered significant.

## Results

The main data acquired are summarized in Table 2.

**Table 2:** Body weight and serum lipids of included patients at consecutive visits (V1. V2. V3).

		**PHT**	**DBS**	**POP**
BM [kg]	V1	77.6 ± 15.1	73.5 ± 15.5	83.9 ± 13.6
	V2	76.9 ± 16.3	77.4 ± 15.7	85.1 ± 12.8
	V3	76.4 ± 16.5	77.8 ± 15.0	84.6 ± 11.3
TG [mg/dl]	V1	98.5 ± 36.6	103.4 ± 60.0	135.2 ± 63.0
	V2	96.6 ± 38.6^‡^	113.1 ± 57.6	150.6 ± 70.3^‡^
	V3	103.1 ± 58.9	117.5 ± 48.4	124.6 ± 51.3
TC [mg/dl]	V1	194.4 ± 36.3^‡^	188.2 ± 34.3^§^	234.3 ± 61.1^‡^^§^
	V2	182.1 ± 34.6	197.6 ± 31.6	204.6 ± 31.8
	V3	187.7 ± 36.2	190.4 ± 32.0	211.3 ± 54.3
LDL-C [mg/dl]	V1	113.9 ± 35.2	111.0 ± 34.7^§^	138.5 ± 40.2^§^
	V2	101.1 ± 31.8	122.4 ± 27.8	121.6 ± 28.1
	V3	111.2 ± 45.8	117.7 ± 30.1	133 ± 46.4
HDL-C [mg/dl]	V1	58.5 ± 17.6	58.5 ± 16.6	56.8 ± 18.7
	V2	61.7 ± 17.4^†^	52.7 ± 17.7^†^	52.9 ± 15.8
	V3	60.9 ± 17.7^†^	49.2 ± 10.5^†^	53.3 ± 18.0

*Statistical significance*:

† *P <0.05 for PHT vs. DBS comparison*;

‡ *P <0.05 for PHT vs. POP comparison*;

§ *P <0.05 for DBS vs. POP comparison*.

Analyses included both the comparison between groups of patients during successive visits and, more importantly, the evaluation of the lipid profile and body weight changes in the course of the therapy in each group.

Although the distribution of body mass was similar in all groups of patients during each visit (V1, V2, V3), the rates of weight change between visits (expressed as a percent of body mass gained compared to the previous measurement) were significantly different between PHT and DBS patients in the first assessed period (−1.0 ± 6.4% vs 5.8 ± 9.7%, *P* = 0.01 for V1–V2 interval). Despite the further decline of the inter-visit body weight variation, the analogical inter-group differences were observed during the longer follow-up (−1.3 ± 8.4% vs. 6.6 ± 11.2%, *P* < 0.01 for V1–V3 interval). From the perspective of the whole study duration, a more subtle difference can also be noticed between the PHT and POP groups with a slight weight gain in the latter one (1.3 ± 4.4%, *P* = 0.04).

In terms of weight gain or loss between visits, regardless of its extent, the groups also varied relevantly. The number of patients who gained weight during the first stage of the study (V1–V2 interval) was significantly higher in DBS and POP than in the PHT group (13 DBS and 11 POP vs. 5 PHT patients, *P* = 0.02 and <0.01, respectively). No intergroup variations were observed in reference to the period V2–V3; nevertheless, over the whole course of the study more patients gained weight in the DBS and POP groups than in the PHT group (12 DBS and 9 POP vs. 5 PHT patients, *P* = 0.05 and 0.04, respectively).

Longitudinal analysis revealed a significant weight gain of 4.3 ± 7.5 kg on average in the DBS group, with a major increase within the first assessed period. Overall body mass in the POP group seemed to be stable; however, a minor increase in the mean body weight between visits V1 and V2 was found.

At the time of inclusion (V1), the average serum levels of triglycerides, total cholesterol, and its fractions were similar in both the PHT and DBS groups, while the concentration of TC in the POP group was significantly higher (for both *P* < 0.01) with the average TC level over the regular value (234.3 mg/dl). Elevated levels of TC were observed in over 90% of patients in the POP group. LDL-C concentrations also varied in the POP group compared to the DBS group (138.5 vs. 111.0 mg/dl, *P* = 0.03) and less significantly to the PHT group (vs. 113.9 mg/dl, *P* = 0.06). During the follow-up, those inter-group variations declined; however, the percentage of patients with an LDL-C level exceeding 100 mg/dl at time point V2 was notably higher in the DBS group than in the PHT group (85 vs. 40%, *P* < 0.01). Simultaneously, the significant differences in the serum concentration of HDL-C for the PHT and DBS groups occurred (61.7 vs. 52.7 mg/dl, *P* = 0.04 upon V2 and 60.9 vs. 49.2 mg/dl, *P* = 0.02 upon V3). The mean plasma level of TG was notably higher in POP than PHT patients at time point V2 (150.6 vs. 96.6 mg/dl, *P* < 0.01), with a higher prevalence of hypertriglyceridemia (57 vs. 15%, *P* < 0.05), without a statistical significance for prior comparisons (Table 2).

**Figure 1:**
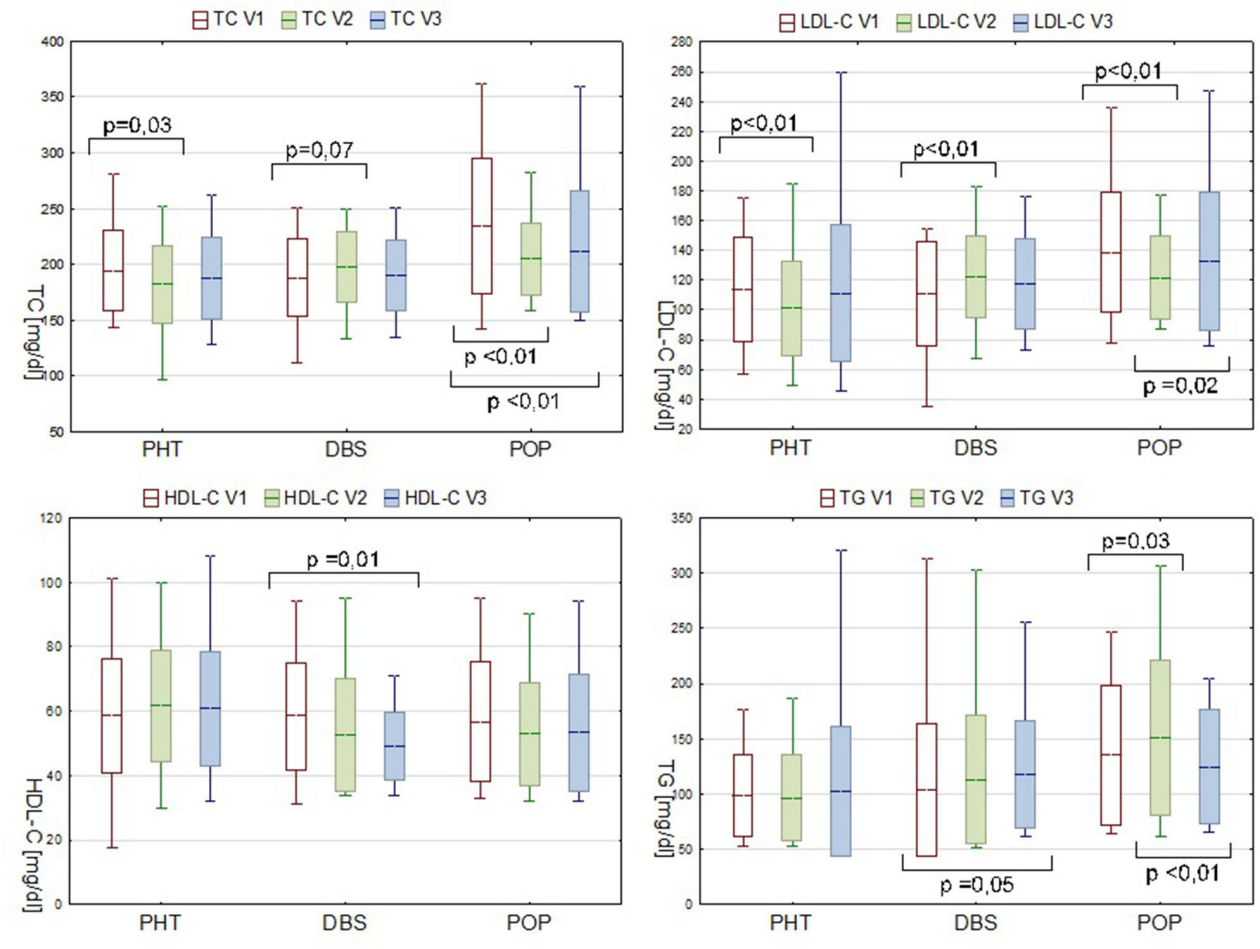
Serum lipids levels in PHT, DBS, and POP groups during consecutive visits. Data are presented as mean ± SD and min—max range.

Analyses were also conducted to evaluate the inter-visit changes in the lipid profile. Between visit one and visit two, substantial decreases in the TC and LDL-C levels were observed in the PHT group (Figure 1).

In the POP group during the first stage of observation, the TG level significantly increased (ΔTG V2-V1 15.4 mg/dl, *P* = 0.03). Concurrent decrease in the serum concentration of TC and LDL-C is probably due to the statin therapy applied, meanwhile, to some of the patients in this group due to the pronounced dyslipidemia diagnosed during the preliminary evaluation (7 out of 14 patients, at some point between V1 and V2, received atorvastatin or simvastatin, dose varying from 10 to 20 mg). In spite of a pharmacological intervention, a secondary increase in the LDL-C level during follow-up was observed (ΔLDL-C V3-V2 7.6 mg/dl, *P* = 0.02) (Figure 1).

The inter-visit analysis of the DBS group patients, which was considered the most valuable in the pre- and postoperative lipid profile comparisons, revealed a gradual decrease in the serum HDL-C level (ΔHDL-C V3-V1−7.7mg/dl, *P* = 0.01). Plasma concentration of LDL-C increased within the V1–V2 interval by 11.4 mg/dl (*P* < 0.01). Although analogical tendency was observed for TC, the result was not statistically significant (*P* = 0.07). Changes in the TG level appeared in a different manner with an alteration only present in the V3–V1 comparison. A recorded rise from 103.4 mg/dl at point V1 to 117.5 mg/dl at V3 was of borderline statistical significance (*P* = 0.05), but nevertheless pronounced, and therefore should not be neglected (Figure 1).

Additional analyses were conducted for the DBS group to estimate the possible direct influence of body mass gain on changes in the lipid profile after surgery. Supplementary division into subgroups based on the weight gain or its lack during a specific time period revealed that significant alteration in HDL-C concerned only the first subgroup (*P* = 0.01 vs. *P* = 0.81 for ΔHDL-C V2–V1 and *P* = 0.02 vs. *P* = 0.24 for ΔHDL-C V3–V1). For other biochemical parameters, no remarkable differences between the patients gaining and not gaining weight were observed.

It is noteworthy that no statistically significant correlation between the quantitative variation in the biochemical parameters and proportionate weight gain was found in the DBS group. In POP patients, there was a strong positive correlation (*R* = 0.704, *P* = 0.01) between the later triglyceride change (ΔTG V3–V2) and the earlier body mass alteration (%ΔBM change V2–V1). Analogically for the PHT group, a moderate positive correlation (*R* = 0.457, *P* = 0.04) between the overall change in the TG level (ΔTG V3–V1) and later body mass variation was observed. Also, the overall alteration of HDL-C (ΔHDL-C V3–V1) was negatively correlated with the body mass change (*R* = −0.460, *P* = 0.04) in this group of patients, but not in the DBS or POP groups.

**Figure 2:**
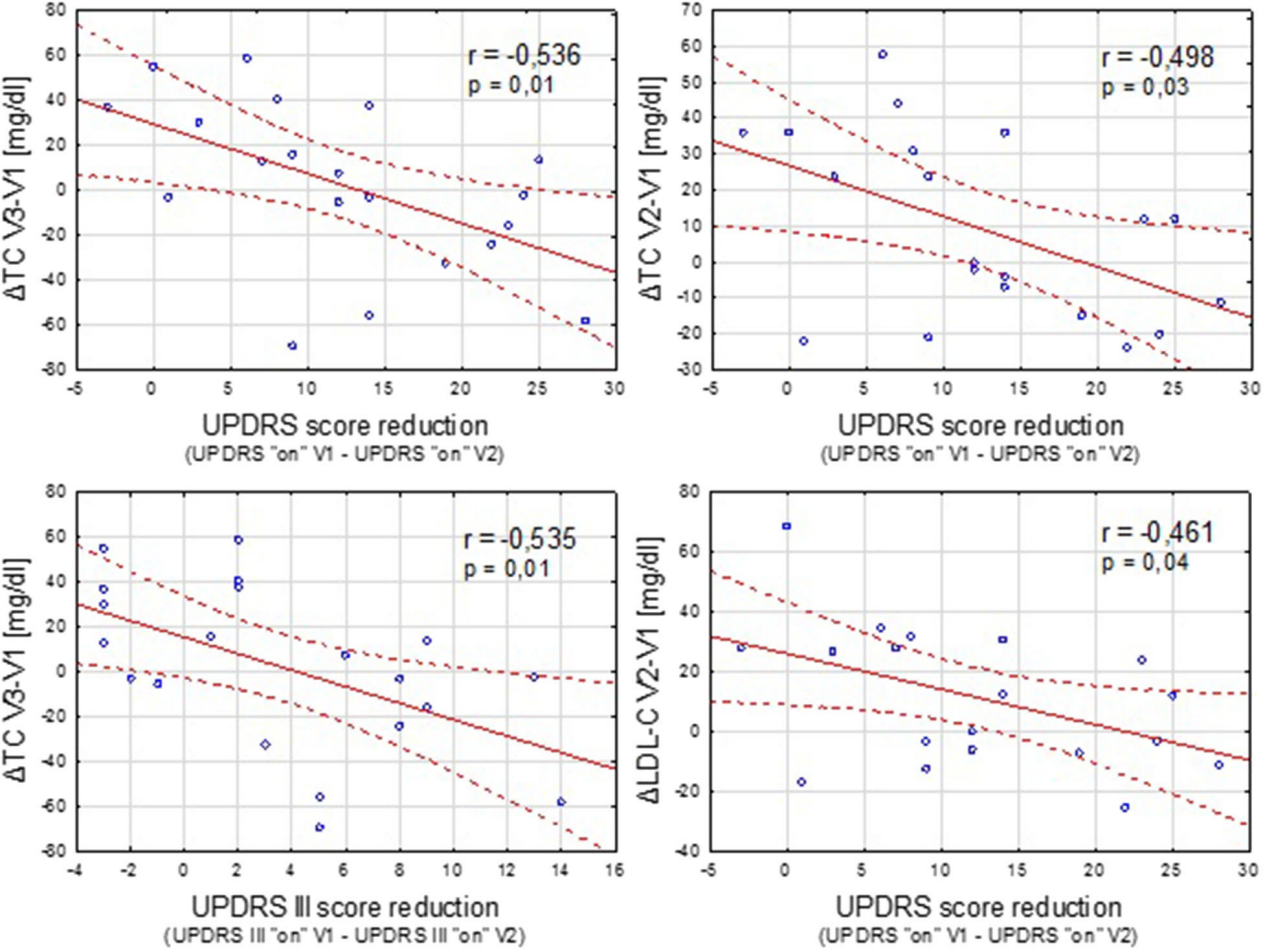
Negative correlation between the clinical improvement after STN-DBS (expressed as reduction in UPDRS “on” and UPDRS III “on” scores between the time points V1 and V2) and increase in the TC and LDL-C concentration.

In the DBS group, we also analyzed the possible relationship between the clinical improvement after the implantation of STN-DBS (expressed as the reduction in UPDRS and UPDRS III scores in “on” condition) and postoperative lipid and weight variations (Figure 2).

## Discussion

This study evaluated some of the factors involved cardiovascular diseases development, depending on the method of the treatment of Parkinson's disease.

Unlike in patients receiving pharmacotherapy alone, in surgically treated patients, the proportionate body weight changes over time were skewed toward more positive values, with major increases within the first months after surgery. Also, the number of patients gaining and losing weight varied between the groups depending on the method of treatment, with a prevalence of body mass increase after DBS. Our results are consistent with other studies implying the substantial rapid body weight gain during the first months after surgery (4, 5, 18). However, in the later follow-up, instead of a further increase in body mass, we observed a tendency to its stabilization at higher than the initial level, which contrasts, for instance, with Foubert-Samier et al. (9).

Contrary to a study conducted by Montaurier (18), our analysis revealed no correlation between the pre-operative UPDRS motor score or post-surgery UPDRS III improvement and the range of body weight gain after STN-DBS. However, sufficient negative correlation was found between the improvement in parkinsonian symptoms after the implantation of DBS and some unfavorable lipid alterations.

Interestingly, negative changes in cholesterol and its fractions were most pronounced during the first months after surgery. At inclusion, hypercholesterolemia was more frequent in the POP group than in the other two groups. In patients with DBS implanted after inclusion in the study, a significant increase of the LDL-C concentration and a similar tendency for TC was noticed already at the first post-surgery visit (V2). Although the HDL-C level decrease during the follow-up in those patients was not significant until the third visit (V3), the inter-group differences between them and the pharmacologically treated individuals became apparent already at V2 time point.

Conversely, the changes in the serum concentration of TG seem to be longer-term. From the perspective of the whole study duration, our investigation revealed an increase of borderline significance in the TG level in patients undergoing surgery within the analyzed period. For patients with DBS implanted prior to our study, analogous alterations in the TG levels were observed during the first assessed time interval. Also, the differences between those patients and pharmacologically treated ones, including a higher percentage of individuals with hypertriglyceridemia (despite the usage of statins), became significant after more than 1.5 years from the surgery.

Simultaneously, our results are in line with past observations indicating a declining tendency in the TC and LDL-C levels in pharmacologically treated patients (11).

As predicted, changes in the lipid profile may be, at least partially, resulting from the body weight gain observed in patients with STN-DBS. We demonstrated that an increase in the TG concentration is correlated with the body mass change for both pharmacologically and surgically treated patients, though not for the individuals within the first 2 years after the implantation of electrodes. Our data also support the statement that the HDL-C level is substantially affected by the body mass change. Surprisingly, no evidence of direct correlation with body weight gain was found for LDL-C or TC.

These results lead to the conclusion that the body weight change is not the only factor inducing the deterioration in the metabolic status of patients with STN-DBS, especially during the first months after the surgery. The underlying mechanisms may be similar to those considered for body weight gain. Since both the PD and DBS affect energy homeostasis (18, 19), their influence on other metabolic processes could be assumed. Analogically to mechanisms suggested by Guimaraes (20) or Markaki (21) for postsurgical body weight gain, the direct effect of local electric current on certain structures adjacent to the subthalamic nucleus and modulation of noradrenergic projections in the brain, involved in the regulation of metabolism, should be taken into account. Another hypothesis is that adverse cardiometabolic alterations may at least partially result from a reduced energy expenditure secondary to motor symptoms improvement after surgery, including abatement of rigidity, tremor, and levodopa-related dyskinesia. In accordance with past observations indicating lower TG and TC levels in plasma of patients receiving levodopa in comparison to untreated individuals with PD (11), the possible side effects of post-surgery levodopa-dose reduction could not be excluded, although no significant correlation with levodopa dose modifications was found for changes in the body weight or lipid profile.

Therefore, a larger population study with a longer follow-up is required to establish direct mechanisms in which DBS may affect the lipid profile and to determine the subgroups of patient especially susceptible to this side effect.

Our results only partially concur with previous reports. Rieu et al. (15) demonstrated a similar correlation between the body mass change and TG level, but observed in a shorter time after the surgery. Lack of such correlation for HDL-C concentration in our surgically treated patients is consistent with their observation. Nonetheless, we found a qualitative relationship between the HDL-C level and body mass gain in the DBS group. The same report also notes a lack of significant changes in the TC and LDL-C levels after surgery, while we demonstrate a significant increase in the concentration of the latter. According to our analysis, the TC level also does not change significantly within 12 months after surgery; however, the comparison of patients with a longer history of DBS and pharmacologically treated patients indicate the occurrence of such alterations. Since the study conducted by Rieu et al. is the only one comprehensively addressing the lipid profile after DBS we know of, the causes of this inconsistency are difficult to determine.

## Conclusion

The most important findings of our study are listed below:

- The post-surgical body weight gain is most pronounced during the first months after the implantation of STN-DBS, with subsequent stabilization at a higher than the preoperative level.
- An increase in the LDL-C concentration is observed early after surgery, followed by adverse changes in the HDL-C and TG plasma levels, which are more gradual; although no significant inter-visit alterations were observed for the TC concentration, the mean TC levels in POP patients fulfilled the criteria for hypercholesterolemia.
- Lipid profile changes after STN-DBS may result from weight gain, but only to some extent—correlation with the body mass change was observed only for TG in the POP group; the lack of such correlation for cholesterol and its fractions in surgically treated patients indicates the existence of other causative factors
- UPDRS score improvement after STN-DBS correlates inversely with the adverse changes in some of the biochemical parameters, probably suggesting a protective role of normalized physical activity secondary to motor melioration.

In conclusion, our data indicate that, alongside its unquestionable efficacy in improving the motor symptoms and quality of life in PD patients, STN-DBS may negatively affect the cardiometabolic profile of the patients.

The observed changes in the lipid profile were not very pronounced; however, even minor increases in the serum levels of TG or LDL-C may play a role in the development of cardiovascular disease. Moreover, according to some publications (22, 23), cardiovascular risk factors potentially contribute to the aggravation of the PD axial symptoms. Therefore, the patients who qualified for STN-DBS, in particular, should have their lipid profile examined regularly and be educated in body weight control and life style modification.

## Data Availability Statement

The raw data supporting the conclusions of this article will be made available by the authors, without undue reservation.

## Ethics Statement

The studies involving human participants were reviewed and approved by Ethics Committee of Medical University of Warsaw. The patients/participants provided their written informed consent to participate in this study.

## Author Contributions

JS-Ć study design, data collection and interpretation, statistical analysis, and writing of the first draft. SS data collection and interpretation, statistical analysis, and manuscript edition. DK study design and organization, data interpretation, and manuscript edition. TM study organization and manuscript edition. AP data management and manuscript edition. AF study design, data interpretation, and manuscript edition. All authors contributed to the article and approved the submitted version.

## Conflict of Interest

The authors declare that the research was conducted in the absence of any commercial or financial relationships that could be construed as a potential conflict of interest.

## Abbreviations

BM: body mass
DBS: deep brain stimulation
HDL-C: high-density lipoprotein cholesterol
LDL-C: low-density lipoprotein cholesterol
PD: Parkinson's disease
TC: total cholesterol
TG: triglycerides
UPDRS: Unified Parkinson's Disease Rating Scale.
